# Expression of E-cadherin and N-cadherin in Epithelial-to-Mesenchymal Transition of Osteosarcoma: A Systematic Review

**DOI:** 10.7759/cureus.49521

**Published:** 2023-11-27

**Authors:** Leo Issagholian, Ethan Tabaie, Akshay J Reddy, Muhammad S Ghauri, Rakesh Patel

**Affiliations:** 1 Medical School, California University of Science and Medicine, Colton, USA; 2 Medical School, California Northstate University College of Medicine, Elk Grove, USA; 3 Ophthalmology, California University of Science and Medicine, Colton, USA; 4 Neurosurgery, California University of Science and Medicine, Colton, USA; 5 Internal Medicine, East Tennessee State University Quillen College of Medicine, Johnson City, USA

**Keywords:** cancer, vimentin, mesenchymal, epithelial, e cadherin, n-cadherin, osteosarcoma

## Abstract

Osteosarcoma (OS) is a debilitating cancer of the bone that commonly afflicts the young and old. This may be de novo or associated with tumorigenic syndromes. However, many molecular mechanisms are still being uncovered and may offer greater avenues for screening and therapy. Cadherins, including E-cadherin and N-cadherin/vimentin, are involved in epithelial-to-mesenchymal transmission (EMT), which is key for tumor invasion. A study reviewing the relationship between OS and cadherins might elucidate a potential target for therapy and screening. A robust literature review was conducted by searching PubMed with the keywords “osteosarcoma”, “cadherin”, “e-cadherin” and “n-cadherin”. Of a preliminary 266 papers, 25 were included in the final review. Review articles and those without primary data were excluded. Loss of E-cadherin is noted in metastatic cell lines of osteosarcoma. Overexpression of E-cadherin or knockout of N-cadherin/vimentin results in loss of metastatic potential. There are several methods of gene knockout, including CRISPR-Cas9 gene editing, viral vector insertion with micro RNA complementary to long noncoding RNA within gene segments, or proteomic editing. Screening for EMT and genetic treatment of EMT is a possible avenue for the treatment of refractory osteosarcoma. Several studies were conducted ex vivo. Further testing involving in vitro therapy is necessary to validate these methods. Limitations of this study involve a lack of in vivo trials to validate methods.

## Introduction and background

Osteosarcoma (OS) is the most common primary malignant tumor of the bone with a global incidence of 3.4 cases per million people annually [1]. OS displays a bimodal age distribution, peaking at 18 and 60 years of age, and is slightly more common in males [2]. OS is highly heterogeneous in nature and is characterized according to location (ie. central, intramedullary, periosteal), degree of differentiation (low- to high-grade), and histopathological differences (ie. conventional, small cell, telangiectatic) [3]. OS most commonly affects long bones of the appendicular skeleton (ie. femur, tibia, humerus) but can also affect the skull and jaw [4]. Central bone tumors are the most common, representing approximately 80% of all OS cases [5]. Despite increased interdisciplinary efforts at treatment, the one-year survival rate remains less than 30% [4].

OS arises from a primitive mesenchymal cellular origin (ie. sarcoma) that undergoes a transformation into a cellular phenotype that exhibits osteoblastic differentiation and production of malignant osteoid [6]. Much work has been done to delineate the complex pathophysiology of this disease, with a focus on cancer stem cells, proteins, and genes that contribute to malignant transformation, proliferation, and the various phenotypes associated with this condition [7]. Several genetic syndromes besides off-target radiation or DNA-damaging agents have been implicated in the increased incidence of developing OS. Examples include familial retinoblastoma (ie. 13q14 inactivates Rb gene), bony dysplasias (ie. Paget’s, fibrous dysplasia, endochromatosis), Li-Fraumeni syndrome (ie. germline TP53 mutation), and Rothmund-Thomson [8,9].

The gold standard for diagnosis is X-ray, whereby appropriate assessment of abnormal pathological destruction, ossification, and chondrocalcification can be performed [10]. Key findings that are highly suggestive of OS are aggressive periostitis (“sunburst configuration”), elevation of periosteum (“Codman’s triangle”), and soft tissue calcification of osteoid matrix (“fluffy clouds”) [10]. MRI can be used for further characterization after initial imaging and is helpful for elucidating the extent of tumor involvement within and outside of bone, including skip lesions. Nuclear medicine (ie. PET, Tc99 MDP) can be done to evaluate highly metabolic lesions, and a final biopsy is performed for staging and characterization of the malignancy [10]. The current mainstay of treatment involves neoadjuvant chemotherapy (ie. doxorubicin, cisplatin, doxorubicin, ifosfamide, and high-dose methotrexate with leucovorin rescue, followed by surgical resection (ie. metastasectomy) and adjuvant chemotherapy [11].

The epithelial-to-mesenchymal transition (EMT) is a process by which epithelial cells acquire mesenchymal characteristics, such as increased mobility and invasiveness. Recent studies have shown that EMT plays a key role in the development and progression of osteosarcomas, a type of bone cancer. Research has shown that EMT is associated with increased expression of mesenchymal markers, such as vimentin and N-cadherin, and decreased expression of epithelial markers, such as E-cadherin, in osteosarcoma cells [2], [12]. A complex web of signaling pathways known to downregulate E-cadherin expression and upregulate mesenchymal markers is involved in mediating EMT [13]. Additionally, new research has emphasized how microRNAs modulate EMT in osteosarcoma [14].

Overall, osteosarcoma is a serious and potentially life-threatening disease warranting prompt diagnosis and effective treatment which are crucial for improving outcomes and preventing the metastatic spread of this disease. A potential strategy for enhancing osteosarcoma treatments is to target EMT. It may be possible to lessen the invasiveness and chemotherapy resistance of osteosarcoma cells by inhibiting the activity of transcription factors that cause EMT or by specifically targeting microRNAs that regulate EMT. The authors of this study aim to conduct a literature review of the recent developments regarding osteosarcoma and the implications of cadherins in the course of this debilitating cancer. Focus is placed on potential as a marker for disease advancement as well as target for therapy.

Methods

**Figure 1:**
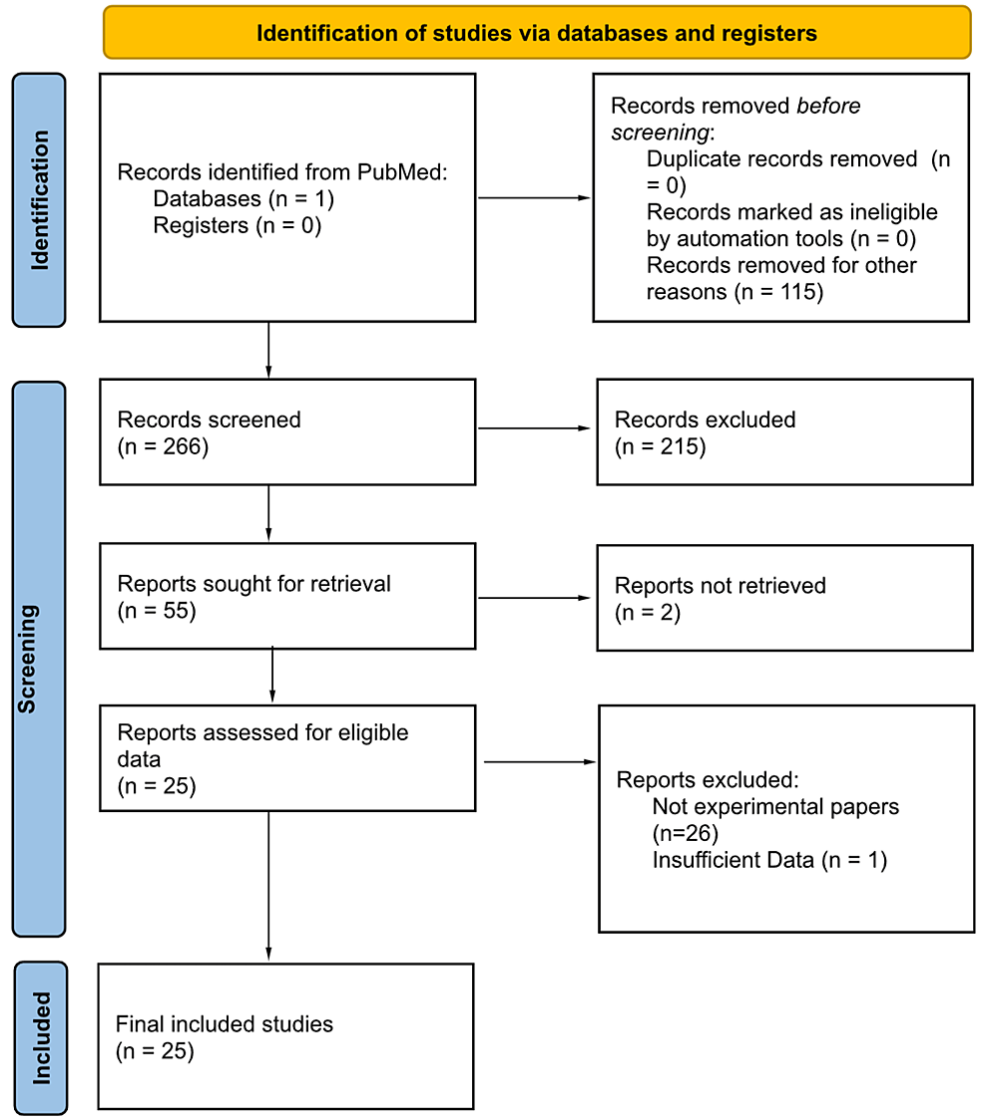
PRISMA diagram illustrating the article selection process. PRISMA: Preferred Reporting Items for Systematic Reviews and Meta-Analyses

The authors performed a literature search using PubMed with the keywords “Osteosarcoma”, “Cadherin”, “E-Cadherin”, and “N-Cadherin” included in the title or abstract of desired publications. The timeframes were closely followed. After the removal of duplicate studies, 266 individual publications remained. 115 studies were excluded on the basis of being outdated and a further 100 lacked full text. 26 studies were then excluded as they lacked the necessary information, adequate description of methods, or limited outcomes. A final 25 studies were included in this review. Data extracted consisted of the gene target of interest, preparation agents used in the cell culture, living and nonliving constituents of cell cultures, method of gene target knockout, the cell line used in the investigation, and the key outcomes of each study. Those studies that did not include at least 2 of the aforementioned data points were excluded. An illustration of the selection process is provided in Figure 1 which follows the guidelines of the Preferred Reporting Items for Systematic Reviews and Meta-Analyses (PRISMA). Statistical analysis of variables was attempted via SPSS Statistics software version 28.0.1.0 (IBM Corp., Armonk, NY) with no notable outcomes.

## Review

The literature search via PubMed yielded a total of 266 studies after the removal of duplicates; 231 studies were excluded as detailed in the methods section. The final 25 studies that were analyzed in this review are listed in Table 1 which includes the target gene of interest along with the primary outcomes listed in each publication. The thorough investigation performed by the authors evaluated each article for potentially analyzable data, however, no such findings were gleaned. Out of the 25 studies investigated, 12 utilized RNA interference to knock out the target genes. Two of the studies included in the 25 utilized patient tumor samples, while the vast majority of experiments were conducted on described osteosarcoma cell lines. There were no standardized cell medium or preparation agents, however, the studies in this review utilized commercially available agents as described in their respective methods. Transfection methods were not universal, however, many studies used premade lipoproteins or customizable lentiviral vectors that were purchased. 

**Table 1:** Current investigative methods of osteosarcoma cell lines and outcomes OS: osteosarcoma; miR: micro RNA; LncRNA: long non-coding RNA; FBS: fetal bovine serum; SDS-PAGE: sodium dodecyl sulfide polyacrylamide gel electrophoresis; PBS: phosphate buffered saline; RPMI medium: Roswell Park Memorial Institute medium; SiRNA: silencing RNA; RNA: ribonucleic acid

Primary author (Year)	Genetic target of interest	Preparation agents	Cell Culture	Method of gene knockout	Osteosarcoma Cell line	Outcome(s)
Chen et al. (2020) [2]	Long noncoding RNA NR_027471	0.3 mg/l G418 100 U/ml penicillin, and100μg/ml streptomycin	Osteosarcoma cell lines (U2OS, Saos-2, MG-63, and HOS), Fetal osteoblast (hFOB1.19), and human bone marrow stem cells (hBMSCs)	miR-8055	Osteosarcoma cell lines (U2OS, Saos-2, MG-63, and HOS)	NR_027471 is significantly downregulated in cancerous. The presence of NR_027471 greatly suppresses osteosarcoma cell viability. Knockout of NR_027471 decreased E-cadherin expression and promoted migration of cells. NR_027471 reduced TP53 inhibition by miR-8055.
Fan et al. (2019) [4]	ROCK1	10% FBS	Human osteosarcoma cell lines HOS, SAOS2, MG-63, U2OS, and OS732 and normal hFOB1.19 osteoblast cells	miR-139, siRNA-mediated silencing	HOS, SAOS2, MG-63, U2OS, and OS732	Knockout of ROCK1 downregulated B-Catenin and p-AKT and upregulated E-Cadherin and p53. miR-139 suppresses the proliferation and invasion of osteosarcoma cells.
Fan et al. (2021) [15]	FAS, GNAS, SCARB1, CXCR4, NF2 and EZR	Phosphate buffer 0.5 mg/mL colchicine 0.075 mol/L potassium chloride	ZOSL-1 hFOB1.19	No knockout	ZOSL-1 cell line derived from U2OS/MTX300 cell line	ZOSL-1, considered a highly metastatic strain, had decreased levels of E-Cadherin expression when compared to other osteosarcoma cell lines.
Gao et al. (2018) [16]	SIRT6	100 U/ml penicillin and 100 μg/ml streptomycin were added to the medium	SAOS-2, MG-63, U2Os hFOB	Silencing RNA against SIRT6 Lentiviral transfection of osteosarcoma lineages with intact SIRT6	SAOS-2, MG-63, U2Os osteosarcoma cell lines	Baseline downregulation of SIRT6 in osteosarcoma lineages. SIRT6 transfected osteosarcoma cell lines had reduced invasion capacity. Several enriched binding sites for SIRT6 were identified on the N-cadherin coding region. Upregulated N-cadherin reduces osteosarcoma.
Habel et al. (2019) [17]	CYR61	12% SDS-PAGE 150 mM NaCl	No axillary cells	Anti-IGF1 neutralizing antibody	murine K7 M2 and human U2OS modified cell lines	IGF1R𝛃 expression is correlated with N-cadherin levels. Silencing of CYR61 reduced N-cadherin expression and reduced IGF1R𝛃.
Huang et al. (2019) [18]	KLF5	50 μg/mL propidium iodine		ML264, a small-molecule inhibitor	U2OS, 143B, and SAOS2	ML264 reduces expression of cyclin E1, cyclin D1, and cyclin-dependent kinase 4. ML264 decreased cancer cell line migration and upregulated E-cadherin.
Huang et al. (2020) [12]	Long noncoding RNA TDRG1	10% PBS	MG-63, U2OS, OS-732 and osteoblast hFOB1.19	Si-TDRG1 group	MG-63 and OS-732	Long noncoding RNA TDRG1 is highly expressed in osteosarcoma cell lines Knockout of TDRG1 showed decreased invasion and migration of osteosarcoma cell lines. Si-TDRG1 transfected cells had increased expression of E-cadherin and decreased expression of N-cadherin.
Ji et al. (2018) [19]	CDH6 (Cadherin-6)	8 mg/mL polybrene; 1 mg/mL puromycin	No auxiliary cells	miR-223-3p	143B and U2OS cell lines	miR-223-3p inhibited osteosarcoma cell line invasion and proliferation. CDH6 inhibition is associated with decreased invasion and proliferation of cancer cell lines. miR-223-3p suppresses CDH6 expression.
Jiang and Huang (2020) [20]	BMIL1	PBS 2 ug/ml	No auxiliary cells	C086+cisplatin	MG-63 cells	C086+cisplatin decreased MG-63 cell lineage invasive and migratory potential. C086+cisplatin decreased expression of BMIL1. E-cadherin is a downstream target of BMIL1.
Jin et al. (2020) [21]	MYH2, MYH7, TUBA1B and TUBB2A	TB (Theabrownin) at 2.13 to 21.3 μg/ml. Crystal violet 1%	No auxiliary cells	Theabrownin on osteosarcoma cells	U2OS cells	TB inhibited the viability of U2OS. TB significantly upregulated the expression of E-cadherin.
Koh and Lee (2020) [22]	c-MET	MMP-7, matrix metalloproteinase-7; PDK1, phosphoinositide-dependent kinase-1; PI3K, phosphinositol-3 kinase; PIP3, phosphatidylinositol (3,4,5)-trisphosphate;	LCN2 knockdown cells	Knockdown of MMP-7 expression	MKN-28 cells	Expression of hepatocyte growth factor reduced expression of E-cadherin. Hepatocyte growth factor is associated with stomach cancer.
Kundu et al. (2021) [23]	Saos 2-ASCs	0.5 mM 1-methyl-3-isobutylxanthine, 1 ng/mL insulin, and 100 μM in- indomethacin) and osteogenic (αMEM supplemented with 10 mM β-glycerol phosphate, 10 mM dexamethasone, and 50 μg/mL ascorbic acid)	osteosarcoma cells cultured	Knockdown of CD44	Human adipose-derived stem cells	EDTA sequesters calcium ions, preventing cell-to-cell interactions between E-cadherins, and disrupting tumor structure.
Li et al. (2019) [24]	GNL3	10% FBS and antibiotics (penicillin, 100 U/mL; streptomycin, 0.1 mg/mL;	MG63, U20S, and normal chondrocyte cells	si-RNA interference	MG63 and U20S	GNL3 is associated with increased tumor survival. Downregulation of GNL3 is associated with increased expression of E-cadherin and decreased epithelial-to-mesenchymal transition.
Li et al. (2019) [25]	DEC1	12% sodium dodecyl, 3% bovine serum albumin, 4% paraformaldehyde,	hFOB, MG63, Saos-2 and U2OS cells	Knockdown of cryptochrome circadian regulator 1	MG63, U2OS, Saos-2	Increased levels of DEC1 upregulated N-cadherin and downregulated E-cadherin. Upregulated DEC1 is found in many forms of cancer, and this study found that DEC1 is found in high levels within osteosarcoma cells, aiding in progression.
Li et al. (2022) [26]	LRIG2	1% protein phosphatase inhibitor, 4% paraformaldehyde	Human OS cell lines (HOS and 143B)	Knockdown LRIG2 expression	Human OS cell lines (HOS and 143B)	Silencing LRIG2 decreases osteosarcoma cell survival. Decreased N-cadherin expression was found in the setting of LRIG2 silencing.
Liang et al. (2019) [13]	LTBP2	90% methanol, stained with 0.1% crystal violet, 20% FBS	MG63 and U2OS cells	miR-421	EMT, MG63 and U2OS cells	Silencing of miR-421, which is found in high concentration in osteosarcoma cells is associated with decreased expression of N-cadherin and increased E-cadherin.
Liu et al. (2020) [14]	Tusc5	75% ethanol, 20% FBS, 4% paraformaldehyde, 0.1% crystal violet	U2OS, Saos-2, MG-63 & HOS	NR_136400 lncRNAs	U2OS cells	Tusc5 deletion or downregulation with long, noncoding RNA NR_13600 is associated with the advancement of osteosarcoma. NR_136400 overexpression is associated with increased E-Cadherin. Decreased NR_136400 is associated with epithelial to mesenchymal transition.
Lou et al. (2021) [27]	circUSP34	4% paraformaldehyde, 1% penicillin	Normal human umbilical vein endothelial cells (HUVEC) and OS cells (KHOS and 143B cell lines) were purchased from the American Type Culture Collection (ATCC).	miR-16-5p	KHOS and 143B cells	CircUSP34 promotes osteosarcoma malignancy and migration. Increased CircUSP34 is associated with decreased E-cadherin and increased N-cadherin.
Ma et al. (2020) [28]	KRAS	10% fetal bovine serum, 10% sodium dodecyl sulfate, 0.1% crystal violet	(HOS, MG63, Saos2 and SJSA1)	MiR-217 was down-regulated	Transfected HOS and MG63 cells	LINC00467 is found in high expression in osteosarcoma cell lines. LINC00467 knockout with si-LINC00467#1 showed decreased expression of N-cadherin and increased E-cadherin.
Miao et al. (2021) [29]	SUV39H2	10% goat serum, 0.2% TX-100, 3% methanol	Human OS cell lines (U2OS, HOS, MG-63), human embryo immortalized osteoblast hFOB1.19	CDH1 was downregulated	U2OS, HOS, MG-63	SUV39H2 is associated with the advancement of osteosarcoma. SUV39H2 overexpression decreased E-cadherin expression and increased N-cadherin expression.
Shabani et al. (2018) [30]	Rac1	N/A	OS cell lines	Knocking down the HMGB1	OS cell lines	Expression of miR-142 is suppressed in osteosarcoma cell lines. Suppression of miR-142 is associated with decreased E-cadherin
Sheng et al (2017) [31]	SPC24	RPMI medium with 10% FBS, 1% crystal violet	143B and U2OS osteosarcoma cells	SPC24 knockdown	143B and U2OS osteosarcoma cells	SPC24 knockdown showed reduced tumor growth in nude mice. Overexpression of SPC24 was associated with increased E-cadherin levels.
Shi et al. (2020) [32]	MSTO2P & PD-L1	10% SDS-PAGE gel	(MG63 and U2OS).	MSTO2P knockdown	MG63 and U2OS	Hypoxia increased tumorigenesis. E-cadherin expression is decreased in the setting of hypoxia. MSTO2P induces expression of downstream products of hypoxia-inducible factor (HIF). Increased expression of MSTO2P is associated with invasion and epithelial to mesenchymal transition of osteosarcoma cells.
Shi et al. (2021) [33]	circPIP5K1A	10% FBS, 4% PFA, 0.2% crystal violet	MG63, 143B, U2OS and Saos2	circPIP5K1A knockdown by siRNA	MG63 and U2OS cell	Inhibition of circPIP5K1A is associated with poor cancer cell viability. Inhibition of circPIP5K1A is associated with increased E-Cadherin expression and decreased invasion of osteosarcoma cell lines.
Tan et al. (2020) [34]	IGFBP3 & MECP2	75% ethanol, 10% FBS, 1% penicillin-streptomycin solution	Human OS cell lines, including MG63, Saos-2, G292, and U20S	miR-384	G292, Saos-2 cells	MiR-384 inhibits osteosarcoma cell line survival and migration. Overexpression of MiR-384 is associated with decreased E-cadherin and increased N-cadherin.

Discussion

The bulk of the results and findings are included in Table 1. E-cadherin is a transmembrane protein that is involved in cell-cell adhesion and plays a crucial role in the maintenance of tissue architecture and organization for the appropriate compartmentalization of basal physiological functions [26]. In the context of osteosarcoma, many studies have attempted to elucidate the relationship between the expression or function of E-cadherin and how it modulates the progression of the disease [35]. Studies have shown that loss of E-cadherin expression is associated with a more aggressive and invasive phenotype in osteosarcoma cells, and it is thought to contribute to the formation of distant metastases [36, 37]. E-cadherin downregulation in osteosarcoma cells has been linked to a number of signaling pathways, including the Wnt and Notch pathways, which are involved in the regulation of cell proliferation and differentiation [36]. In addition, E-cadherin has been shown to play a role in the regulation of the EMT [38]. In osteosarcoma, decreased E-cadherin expression has been associated with an increase in the mesenchymal phenotype of tumor cells, which contributes to the progression of the disease [36]. Overall, the role of E-cadherin in osteosarcoma progression is complex and still not fully understood, but it is clear that decreased expression or function of E-cadherin contributes to the invasive and aggressive behavior of osteosarcoma cells [26]. Thus, E-cadherin may be a potential target in the development of novel therapeutic strategies for the treatment of osteosarcoma.

Targeting of N-cadherin or vimentin by inserted micro RNAs (miRNAs) or silencing RNAs (siRNAs) yields a similar result to increased expression of E-cadherin [16]. Gao et al. elucidated that the SIRT6 gene, which is often suppressed in osteosarcoma cell lines, downregulates N-cadherin [16]. Similarly, Habel et al. found that a gain-of-function mutation in the CYR61 gene, commonly found in mesenchymal progenitors, promoted N-cadherin expression and metastatic potential when placed in murine models [17]. The expression of N-cadherin is inversely proportional to E-cadherin expression and the downregulation of N-cadherin leads to more E-cadherin and a less invasive tumor phenotype [16, 17]. Therapeutic targeting of N-cadherin expression or further upstream targets, possibly by transfection with siRNA or miRNA as detailed in vitro by the authors mentioned in Table 1, may help slow the progression of osteosarcoma or possibly serve as an adjunct to primary therapy to mitigate metastasis.

According to the findings that were gleaned from the body of research that was used to compile Table 1, the most often utilized cell lines included U2OS cells. Because U2OS cells originated from a human osteosarcoma tumor [32], they have many of the same characteristics and mutations as the tumors of actual patients. As a result, these cells are the most suitable kind of cells for the purposes of conducting research and analyzing the results of that research. In addition to this, it has been discovered that U2OS cells react well to treatment [31], which means that clinicians can utilize these cells to forecast how true osteosarcoma tumors would respond to novel treatments and procedures. Of the chosen cell lines, MG-63 cells were the type of cell that occurred the second most frequently. The MG-63 cells, just as the U2OS cells, were derived from a human osteosarcoma tumor [32]. This fact may help to explain why researchers decided to use the MG-63 cells to investigate the role that cadherin plays in osteosarcoma. In addition, it is possible that these cells were chosen since it is well-documented that they may produce a mineralized extracellular matrix in a manner that is analogous to the activities of osteosarcoma cells in vivo. Standardization of cell lines aids in identifying clear patterns and consistent outcomes as demonstrated in the studies involved in this review. When considering these cell lines and interpretation of results, it does limit the translation to in vivo where patient tumors are not standardized, and there may be little to no overlap in expression profiles outside of a common progenitor status. Interpretation of results should be viewed through this lens.

The authors listed in this study utilized multiple methods of transfection and insertion of genes. Lipofectamine series 2000 and 3000 have been listed as a common method which includes a lipid vector and a CRISPR component for transcript editing [2, 4]. A lentiviral vector was also used with puromycin resistance for cell selection [14, 16, 27]. Luciferase assay with radioactive ligands can be used to confirm the expression of desired plasmid products [2, 4]. Studies used western blot analysis to confirm the expression of protein end products such as E-cadherin, N-cadherin, and p53 levels [16]. Relative uniformity in some of the methods employed by in vitro experiments across studies aids in the collective interpretation of similar results. However, many of the matrix solutions, purification methods, and co-cultures did vary, which limited any statistical analysis across multiple studies listed in Table 1.

While the majority of studies were in vitro, Huang et al. evaluated tumor samples from 87 patients with confirmed osteosarcoma for the expression of TDRG1 expression, and Ji et al. evaluated samples from 133 patients in the evaluation of CDH6 gene [12, 19]. Huang et al. evaluated TDRG1 gene expression in tumor cells from 87 patients with confirmed osteosarcoma. Elevated levels of TDRG1 gene product had lower E-cadherin expression and in patients whose cells had high levels of expression, their tumors were staged higher, had higher grades, and were larger in size [12]. While the study by Huang et al. was not a therapeutic evaluation, the methods displayed the potential for monitoring and detection of genes expressed by osteosarcoma and targeting them directly with siRNA as the authors did with good in vitro results [12]. The authors did not implement a viral or lipoprotein vector for in vivo treatment of these 87 patients with si-TDRG1 as a proper method of implementation has yet to be described, as well as concerns with viruses who insert their genomes such as off-target insertion [12]. Similarly, Ji et al. evaluated 133 patient tumor samples with confirmed osteosarcoma in the evaluation of the CDH6 gene. High levels of CDH6 in patient tumors were associated with greater size, later staging, poorer differentiation, metastasis, and a greater ratio of N-cadherin to E-cadherin [19]. The translational evidence presented in both in vitro studies from described cell lines like MG-63 and U20S as well as in studies that included tissue samples from patient tumors supports the thought that the results from these molecular experiments hold weight as a potential lead in the discovery of a novel treatment for osteosarcoma.

The migratory potential of tumor cell lines is displayed by the EMT, wherein complex signals and deleterious mutations drive differentiated epithelial cells to regress and become more like their invasive progenitors [39]. In the context of osteosarcoma, the transition to invasive forms allows osteoblast progenitors to invade hematogenously and seed elsewhere which is termed malignancy [40]. Several of the studies presented in this article displayed that modification of cadherin expression, often upregulation of E-cadherin and downregulation of N-cadherin, resulted in a decreased rate of EMT and subsequent decreased malignant potential of osteosarcoma cell lines in vitro [36, 40, 41]. Micro RNAs (miRNAs), which are complementary to the desired mRNA to be destroyed, were commonly employed by researchers in knocking down or silencing expression of select genes whose downstream products included N-cadherin, vimentin, or in upregulation of E-cadherin [2, 4, 13, 19, 27, 28, 34]. The expression of E-cadherin was closely inversely related to the expression of N-cadherin, which predicted the malignant potential and invasiveness of osteosarcoma cell lines [27-29]. Several studies in this article concluded that upstream modification of E-cadherin expression can determine a tumor cell line’s invasiveness and is associated with the severity of cancer and tumor cell survival [24-26].

The expression of N-cadherin and loss of expression of E-cadherin can be regarded as the point of progression to malignancy of an oncogenic cell line [42]. The use of nucleotide amplification assays such as polymerase chain reaction (PCR) has been employed in the evaluation of the level of N-cadherin expression in the investigation of a tumor for its malignant potential [43]. PCR analysis performed on E-cadherin levels of newly diagnosed leukemia patients found that lower levels of E-cadherin mRNA on real-time PCR (RT-PCR) were associated with greater severity of malignancy [41]. Fragments of E-cadherin have been identified in bacteria-related malignancies, suggesting that the breakdown of normal cell adhesion molecules by bacteria contributes to the tumorigenicity of epithelial cell lines [44]. Previous studies have utilized a surrogate measurement of E-cadherin and N-cadherin by evaluating tumor expression of miRNAs, long noncoding RNAs (lnRNAs), exosomes, or other RNA fragments, however, direct serum measurement of E-cadherin may offer greater insight on malignancy potential than upstream effector molecules [9, 45]. This vastness of RNA components affecting gene expression has been termed the tumor competing endogenous RNA (ceRNA) [14]. Interruption of the tumor ceRNA leads to the desired phenotype of experiments with reduced invasiveness and growth of osteosarcoma cells [14]. Interestingly, Liu et al. utilized miRcode to predict what their lncRNA potentially targeted in tumor cells [14]. This strategy could be employed to elucidate how the illusive ceRNA functions. Further studies exploring the entirety of tumor ceRNA may be insightful in the treatment of osteosarcoma. Lou et al. explored how circUSP34, a circular RNA, prevents miRNA from binding to their effectors, allowing for tumor cells to downregulate E-cadherin [27]. The various functions and forms of RNA are currently poorly understood and are potential targets of therapy.

P53 or TP53 is an important regulator protein in the cell cycle which promotes senescence with loss of expression in the setting of invasive osteosarcoma [2]. Upregulation of upstream products of the E-cadherin gene including messenger RNA, micro RNA, and transcription factors like long noncoding RNA not only increased the expression of E-cadherin and decreased the expression of N-cadherin, but also increased the expression of p53 [2]. Further investigations into the relationship between p53 and E-cadherin expression by Fan et al. displayed that knockout of the ROCK1 gene whose end products inhibited E-cadherin expression also increased levels of p53 [4]. While these findings may suggest a direct relationship, it is more likely that genes involved in the downregulation of E-cadherin have broader roles such as promoting stemness of cells. By downregulating p53, these genes serve to promote stemness, and in conjunction with E-cadherin loss, allow for the migration and replication of tumor cells, which lose the ability to suppress such tumorigenic genes.

E-cadherin expression modification has been explored as a target for therapy in multiple disease processes. In the results, multiple in vitro studies displayed a decrease in tumor malignancy and invasive potential with an increase in E-cadherin expression and a decrease in N-cadherin expression via transfection with silencing or micro RNAs targeted to upstream effectors [2, 12, 16, 20, 24, 25, 33, 34]. Bi et al. utilized an in vivo murine model to transfect endometrial cells with homeobox proteins that resulted in downstream upregulation of E-cadherin, improving embryo adhesion and implantation [46]. Interestingly, in tumors with a strong epithelial layer concealing the inner mass, disrupting E-cadherins allowed immune cells to infiltrate and target tumor cells [23]. This principle was also applied in the investigation of using ethylenediaminetetraacetic acid (EDTA) in disrupting cadherins of glioblastomas in the brain and may be worthwhile investigating within the bone [47]. Yan et al. showed that post-myocardial infarction mice that were transfected with N-cadherin adenovirus were able to better cope with and protect against ischemia via mesenchymal stem cell activation [35]. The concern would be the potential for malignancy with overexpression of N-cadherin, however, controlled upregulation may have therapeutic benefits [17]. Further investigation into cardiac remodeling and the aid offered by EMTs may elucidate stem cell exploiting methods for post-MI recovery.

## Conclusions

Loss of E-cadherin expression is integral to the EMT in OS cell lines. The expression of E-cadherin is tightly regulated by multiple genes and their inhibitory RNA. These genes are modulated by both promoting and inhibiting signals that may take the form of miRNA, siRNA, lncRNA, and circRNA. Insertion or deletion of these RNA elements as well as whole gene suppression has been achieved in vitro by the use of viral or lipoprotein vectors with a CRISPR component. This represents an avenue for therapy with advancements in gene editing. Studies have shown promising downstream results of E-cadherin upregulation including decreasing malignancy of OS cell lines, reduction in murine tumor size, and return to epithelial differentiation of cancer cells. Further investigation and formulation are needed to validate these in vitro methods.
